# Mechanisms of Yajieshaba in the treatment of liver fibrosis through the Keap1-Nrf2 signaling pathway

**DOI:** 10.3389/fphar.2023.1124015

**Published:** 2023-05-09

**Authors:** Yuanmei Bai, Haimei Wu, Lijie Zheng, Yuhuan Xie, Feifan Liu, Yan Wan, Qiongchao Li, Peixin Guo

**Affiliations:** ^1^ College of Ethnic Medicine, Yunnan University of Chinese Medicine, Kunming, Yunnan, China; ^2^ The Second Affiliated Hospital of Guangzhou University of Chinese Medicine, Guangzhou, China

**Keywords:** Yajieshaba, liver fibrosis, Keap1-Nrf2 oxidative stress pathway, pharmacodynamics, mechanism

## Abstract

Yajieshaba **(**YJSB), a traditional Dai medicine formula containing botanical drugs, is commonly employed in Yunnan due to its significant therapeutic effects on liver protection. Consequently, to determine the efficacy of YJSB and the mechanism of action of Kelch-like ECH-associated protein 1 (Keap1)-nuclear factor erythroid 2-related factor 2 (Nrf2) pathway against liver fibrosis. We wanted to see if YJSB could treat CCl_4_-induced liver fibrosis by regulating the Keap1-Nrf2 signaling pathway. YJSB significantly improved liver function biochemical indices, liver fibrosis quadruple, hydroxyproline (Hyp), and transforming growth factor-β1 (TGF-β1) levels. The staining results demonstrated that the degree of liver fibrosis was significantly reduced. YJSB reduced the content of malondialdehyde (MDA) and elevated the content of superoxide dismutase (SOD) in the liver, exhibiting antioxidant effects; meanwhile, it regulated the expression of Keap1-Nrf2 pathway protein, increased the expression of NAD(P)H: Quinone oxidoreductase (NQO1), Heme Oxygenase 1 (HO-1), Glutamate cysteine ligase modifier subunit (GCLM), and Glutamate cysteine ligase catalytic subunit (GCLC) expression in the liver decreased while Nrf2 expression increased. Fluorescence immunoassay studies demonstrated that YJSB promoted the trans-nuclearization of Nrf2. YJSB possesses anti-liver fibrosis pharmacological effects that improve liver function and effectively counteract CCl_4_-induced liver fibrosis damage. The mechanism of action might be related to the regulation of protein expression of the Keap1-Nrf2 pathway, increasing the ability of the body to resist oxidative stress and reduce oxidative stress injury.

## 1 Introduction

Liver fibrosis is a pathological process of “damage-repair” of the liver stimulated by a chronic viral infection, alcohol abuse, drugs, toxic substances, autoimmunity, gallbladder disease, and other factors (Lutz et al., 2020). During this process, hepatic stellate cells proliferate and stimulate the deposition of collagen-based extracellular matrix (ECM), causing abnormal liver function, inducing liver fibrosis, and promoting the formation of cirrhosis, which is estimated to affect 1%–2% of the global population and cause over 1 million deaths annually, severely affecting people’s quality of life (Higashi et al., 2017). Currently, the primary treatment for liver fibrosis is antifibrotic therapy and treatment for the underlying cause. For instance, colchicine is employed to prevent the secretion of pro-collagen molecules (Tingting et al., 2020). However, these medications have significant adverse effects, and colchicine could lead to renal damage that results in hematuria and oliguria (Du et al., 2021). In contrast, ethnomedicines possess multi-target, multi-pathway, and multi-level neuroprotective effects and have become popular in recent years in the treatment of liver fibrosis (Rong et al., 2021).

According to Dai medicine, liver fibrosis is classified as liver disease, which is a clinical symptom of toxicity that causes the organism to malfunction (Bai et al., 2022). YJSB is a common medication employed by the indigenous Chinese Dai people to treat liver disease. It has been employed in Dai medicine clinics for over 2,000 years to cure hepatitis and cirrhosis induced by alcohol, drugs, or toxic substances (Xiaohua et al., 2015; Yang et al., 2022). In this composition, *Arundina graminifolia* (D. Don) Hochr, *Dregea sinensis* Hemsl, *Fibraurea recisa* Pierre, *Pueraria montana* var. *lobata* (Willd.) Maesen & S.M.Almeida ex Sanjappa & Predeep, and *Mappianthus iodoides* Hand.-Mazz are the principal botanical drugs (Table 1); *Anodendron nervosum* Kerr, *Clerodendrum chinense* (Osbeck) Mabb, and *Glycyrrhiza uralensis* Fisch. ex DC are the supplementary botanical drugs (Table 1). The entire composition is applied in the ratio 14: 5: 7: 5: 6: 18: 15: 20. While jatrorrhizine (C_20_H_20_NO_4_) and berberine (C_20_H_18_NO_4_^+^) are the primary alkaloid components in YJSB (Xiaomei et al., 2022). Some experimental studies discovered that YJSB could reduce the content of enzymes, such as alanine aminotransferase (ALT) and aspartate aminotransferase (AST) in the serum of mice with carbon tetrachloride CCl_4_-induced liver injury and exhibit hepatoprotective effects (Xiaohua et al., 2015). Meanwhile, a previous study discovered that in a rat model of liver injury induced by acetaminophen (APAP), YJSB could reduce the serum levels of malondialdehyde (MDA) and lactate dehydrogenase (LDH) and increase the levels of superoxide dismutase (SOD) and plasma glutathione peroxidase (GSH-Px), which would exert an antioxidant effect.

**TABLE 1:** Contents of YJSB decoction.

Local name	Species name	Family name	Drug name
Weng shang hai	*Arundina graminifolia* (D. Don) Hochr	Orchidaceae	Arundinae herba
Dai bai jie	*Dregea sinensis* Hemsl	Apocynaceae	Dregeae radix
Hei tao han	*Fibraurea recisa* Pierre	Menispermaceae	Fibraureae caulis
Hebie	*Pueraria montana* var. *lobata* (Willd.) Maesen and S. M. Almeida ex Sanjappa & Predeep	Fabaceae	Puerariae lobatae radix
Deng hei han	*Mappianthus iodoides* Hand.-Mazz	Icacinaceae	Mappianthuse caulis
Jie long meng la	*Anodendron nervosum* Kerr	Apocynaceae	Anodendrone caulis
Bai hua chou mu dan gen	*Clerodendrum chinense* (Osbeck) Mabb	Lamiaceae	Clerodendrume radix
Sha yin	*Glycyrrhiza uralensis* Fisch. ex DC	Fabaceae	Glycyrrhizae radix et rhizoma

All botanical drugs were obtained from Xishuangbanna Dai Nationality Hospital in Yunnan Province, China.

In recent years, oxidative stress has been recognized as a significant contributor to liver fibrosis (Yan et al., 2019). According to Yan et al. (2019), overexpression of antioxidants or antioxidant genes could prevent the activation of hepatic stellate cells and exert antifibrotic effects. The Keap1-Nrf2 signaling pathway is an important mechanism in the oxidative stress response; it regulates the expression of several antioxidants and detoxifying enzymes and plays an important role in maintaining the body’s redox homeostasis (Yanshuang et al., 2020). Vincenzo and Laura (2018) discovered that knocking down Nrf2 induces hepatic stellate cells activation, as shown by an increase in α-SMA-positive cells and by gene expression induction of ECM components (collagens and fibronectin). Reduced Nrf2-levels in hepatic stellate cells resulted in increased migration and decreased proliferation. Furthermore, they found that activation of Nrf2-deficient hepatic stellate cells was linked to TGF-β1 activity.

At present, the anti-hepatic fibrosis mechanism of YJSB remains unclear. This study, CCl_4_ was administered intraperitoneally to induce a liver injury model in rats, resulting in inflammation, oxidative stress, and fibrosis. It was discussed whether the mechanism of YJSB against liver fibrosis was by regulating proteins in the Keap1-Nrf2 pathway, enhancing the protective effect on liver cells, reducing the production of oxidative stress factors, and thereby inhibiting the process of liver fibrosis.

## 2 Materials

### 2.1 Experimental animals

A total of 56 male Sprague−Dawley (SD) rats (200 ± 20 g) were obtained from the Animal Center of Kunming Medical University. The animal certificate number was (Yunnan) K2015–0002. The Yunnan University of Chinese Medicine’s Ethics Committee reviewed and approved the animal study (Ethical number: R-06202012). The rats were fed, watered without restriction, and maintained in a favorable environment (23°C ± 2°C, 45%–55% humidity) with a 12:12 light−dark (LD) cycle.

### 2.2 Drugs

All the botanical drugs of YJSB were procured from Dai Hospital in Xishuangbanna, Yunnan Province, China, and authenticated as genuine by Professor Yanfang Lin, the chief expert in Dai medicine (Table 1). Shanghai Yuanye Biotechnology Co., Ltd. Provided the positive controls for the study, namely, compound Biejia-Ruangan tablet (CBRT) and colchicine (Col) (China, Batch No.: 20200402, S18047). Tianjin Wind Ship Reagent Technology Co., Ltd. Supplied the CCl_4_ (China, Batch No.: 20180301). Olive oil was obtained from Meilunbio (United States, Batch No.: MB13084). Jatrorrhizine and berberine were acquired from Shanghai Yuanye Biotechnology Co., Ltd. (China, Batch No.: Z11M8S35796, Y18N8S49558).

### 2.3 Reagents

Nanjing Jiancheng Biological Engineering Research Institute Co., Ltd. Provided serum glutamic pyruvic transaminase (GPT), serum glutamic oxaloacetic transaminase (GOT), SOD, and MDA kits (China, Batch No.: C009–2–1, C010–2–1, A001–3, and A003–1–2). PIIINP, ColIV, LN, and HA kits were purchased from Jiangsu Enzyme Standard Biotechnology Co., Ltd. (China, Batch No.: MB-1580A, MB-7163A, MB-1837A, and MB-2052A). GCLC, GCLM, NQO1, and HO-1 were obtained from Shanghai Jining Biotechnology Co., Ltd. (Batch No.: 202005). Goat anti-rabbit IgG-488 and goat anti-mouse IgG-594 were purchased from Proteintech (United States, Batch No.: SA00013–2, SA00013–3).

### 2.4 Instruments

Thermo Scientific Inc. Provided the enzyme marker (Varioskan Flash) and the low temperature high speed centrifuge (Primo R) (United States). Shanghai Hengke Instruments Co., Ltd. Provided a constant temperature drying oven (China). Agilent Technologies Inc. Provided an analytical liquid chromatograph (Agilent 1,200) column C18 (250 mm × 4.6 mm, 5 µm) (United States). An automatic digital pathology section scanner (KF-PRO-005-EX) was obtained from Ningbo Jiangfeng Bioinformatics Co., Ltd. (China). Besides, a nitrogen purge instrument (NDK200–2) was obtained from Mio Instruments (China).

## 3 Methods

### 3.1 Drug preparation

Preparation of Yajieshaba (YJSB) low-dose, middle-dose, and high-dose solutions: Prescription botanical drugs were extracted 12 times with water, twice for 20 min each time, and then mixed with YJSB aqueous extract and filtered through gauze. Double-distilled water was added to prepare a solution of 0.44 g botanical drug/mL as the YJSB high-dose solution, and the middle-dose and low-dose were obtained by dilution in equal proportions to 0.22 g raw drug/mL and 0.11 g raw drug/mL as the YJSB middle and low dose solutions with double-distilled water. Colchicine (COL), as an inhibitor of cellular microtubule polymerization, can inhibit cell division and proliferation, as well as the synthesis and secretion of the extracellular matrix, such as collagen, in connective tissue cells. It inhibits the inflammatory response, improving the degree of CCL_4_-induced liver fibrosis and patient survival with few serious side effects (Xia et al., 2006; Zesong et al., 2006; Jian-Chang et al., 2009). COL solution preparation: COL powder is weighed with a precision electronic balance, dissolved in double-distilled water, prepared into 0.027 mg/mL colchicine solution, and stored at 4°C. The compound Biejia-Ruangan tablet (CBRT) may prevent liver fibrosis by regulating the expression of inflammatory factors in the body, primarily by down-regulating pro-inflammatory factors and up-regulating anti-inflammatory factors, increasing matrix metalloproteinase inhibitory factor activity and promoting the activation and proliferation of hepatic stellate cells. This can effectively prevent hepatocyte damage and the pro-fibrotic effects of inflammatory factors in the body (Zesong et al., 2006; Yonghong and Chuan, 2022). To make the CBRT solution, a soft liver tablet of turtle shell was dissolved in double-distilled water to make a 0.054 g/mL solution, which was stored at 4°C. The solution of 40% CCl_4_ was made by mixing olive oil and CCl_4_ solution in a 6:4 volume ratio.

### 3.2 Analysis of the phytochemical components in blood from YJSB-treated rats by high-performance liquid chromatography (HPLC)

The liver injury model was replicated in male healthy SD rats and randomly divided into a normal group and a drug group of 6 rats each, with the normal group gavaged with an equal volume of double-distilled water and the drug group gavaged with YJSB 2.2 g/kg (Yang et al., 2022), both in a volume of 10 mL/kg. At 1 h after the gavage, blood was taken from the abdominal aorta and centrifuged. The supernatant was taken in a centrifuge tube; five times the amount of methanol was added, vortexed for 10 min, then centrifuged at 6,000 r/min for 15 min. The supernatant was taken, the solvent was evaporated under nitrogen at room temperature, 400 µL of methanol was added to dissolve, and the sample was filtered through a 0.22 µm filter tip and analyzed. The drug-containing serum, normal serum, and control received similar treatments. On a ZORBAX Eclipse Plus-C18 column (250 mm × 4.6 mm, 5 µm), the chromatographic separation was performed using a mobile phase of acetonitrile and 0.1% phosphoric acid solution (22:78) and 50 µL of triethylamine per 1,000 mL of 0.1% phosphoric acid solution at a flow rate of 1 mL/min and a detection wavelength of 346 nm. The injection volume was 80 µL.

### 3.3 Animal modeling and treatment

**FIGURE 1:**
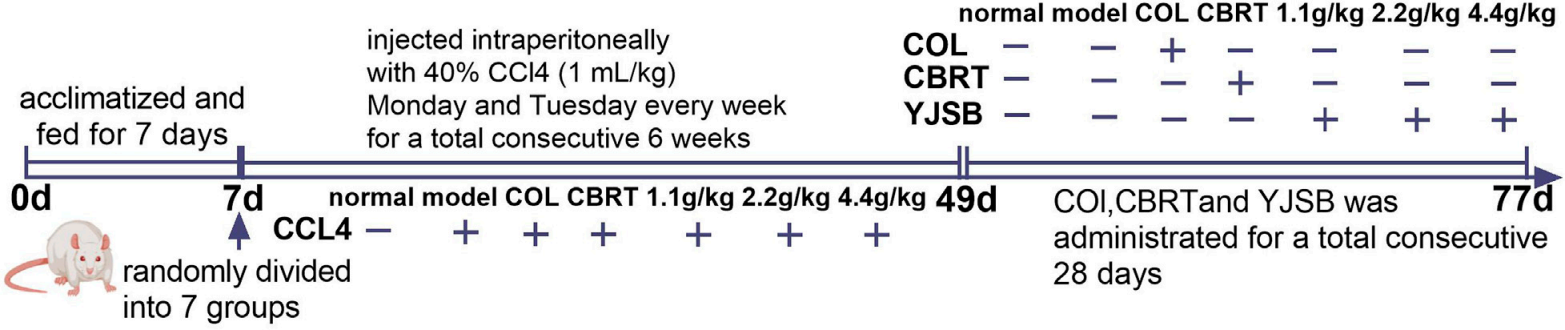
Timeline in the experimental intervention.

Healthy male SD rats were acclimatized and fed for 1 week before the test and randomly divided into seven groups of eight rats each, namely, the normal group, the model group, the Col group, the CBRT group, and the YJSB low, middle, and high dose groups. Except for the normal group that was not intervened, the remaining groups were injected intraperitoneally with 40% CCl_4_ olive oil solution at a rate of 1 mL/kg each for model replication. The injections were scheduled for Monday and Tuesday every week for two consecutive days: 1 time/day for 6 weeks (Figure 1). After the model was finished, the normal and model groups were gavaged with double-distilled water. The YJSB group gavage doses were 1.1 g/kg, 2.2 g/kg, and 4.4 g/kg for the YJSB low, middle, and high dose groups, respectively (earlier studies revealed that the maximum single dose of YJSB mice was 88 g raw drug/kg. Therefore, 1/80, 1/40, and 1/20 of the maximum dose were selected as the low, middle, and high doses of YJSB, respectively). In addition, 0.54 g/kg for the CBRT group and 0.27 mg/kg for the Col group were administered 1 time/day and continued for 4 weeks (Figure 1). The medication dose was determined following the conversion formula and the clinical daily dose.

### 3.4 Determination of liver function and biochemical parameters

After the last administration, the 12-h fasted rats were weighed and anesthetized with pentobarbital (150 mg/mL), followed by blood sampling from the abdominal aorta. The sample was coagulated at room temperature for 30 min and subjected to centrifugation at 4°C, 4,000 rpm for 10 min. The serum was extracted from the sample following centrifugation and was employed to detect AST, ALT, ALP, TBIL, PIIINP, ColIV, LN, HA, Hyp, and TGF-β1.

For pathological examination, the right lobe of the liver was removed and fixed in 4% neutral formaldehyde. The remaining liver tissues were stored in the refrigerator at −80°C for subsequent usage.

### 3.5 H&E, Masson, Ag staining

The liver tissues were fixed in 4% paraformaldehyde solution for 24 h, following which paraffin sectioning, dewaxing, and dehydration were performed, and H&E, Ag, and Masson’s trichrome staining were performed, respectively. The histopathological observation of the liver was performed using an automatic digital pathological biopsy scanner, and the changes in hepatic inflammatory infiltration, collagen fiber, and reticular fiber in each group were assessed.

### 3.6 Determination of oxidative stress factors

In the liver tissue homogenates, the contents of SOD and MDA were measured following the instructions of the kit.

### 3.7 Elisa method to determine GCLC, GCLM, NQO1, HO-1 protein content

The protein concentration of liver homogenates was determined using a bicinchoninic acid (BCA) kit, and the expression of GCLC, GCLM, NQO1, and HO-1 was determined using an ELISA kit.

### 3.8 Western blot method to determine Keap1, Nrf2 protein expression

Total protein was extracted from liver tissue, followed by the following steps: gel preparation (10% isolate, 5% concentrate); constant pressure electrophoresis (70 V, 30 min followed by 110 V, 2 h); constant pressure membrane transfer (110 V, 1 h); milk closure at room temperature for 2 h; Keap1 (1:500), Nrf2 (1:1,000), GAPDH (1:15,000), and primary antibodies were closed overnight at 4°C in the refrigerator; rabbit anti-rat secondary antibodies (1:20,000) closure at room temperature for 1 h and developed. The expression of the target protein was assumed to be represented by the ratio of the target protein bands to the optical density of the internal reference protein bands.

### 3.9 Fluorescence immunoassay to determine Nrf2 protein in the nucleus

The liver tissue was fixed with 4% paraformaldehyde overnight, dehydrated by an automatic dehydrator, embedded in paraffin, sliced, dewaxed and hydrated, then stained by conventional HE, sealed with neutral gum, observed, antigen repaired, painting circle and serum closure (Wang, 2017), where the antibodies Keap1 and Nrf2 were incubated at a concentration of 1:500 and 1:200, respectively. Keap1 and Nrf2 protein expression and the entry of Nrf2 into the nucleus were observed under the fluorescence microscope.

### 3.10 Statistical methods

For statistical analysis, SPSS 21.0 software was used, and the data of each group were expressed as “mean ± standard deviation” (x ± s), and the one-way ANOVA was used for the two-way comparison between multiple groups. The difference was statistically significant with a *p*-value of <0.05.

## 4 Results

### 4.1 Quality control and major chemical composition of YJSB

First, YJSB is a hospital preparation developed by Xishuangbanna Dai Nationality Hospital in Yunnan Province, China. It has been made into capsules (also known as Baijie capsules) and was approved by Yunnan Food and Drug Administration in 2008 (approval number: Yunnan Pharmaceutical Production (Z) 20082252K). There are corresponding quality standards and specifications for hospital preparations to ensure the stability of their components (Table 2). All the medicinal materials were purchased from Xishuangbanna Dai Nationality Hospital of Yunnan Province, and the content of active ingredients met the requirements of Chinese pharmacopoeia and local laws and regulations. For example, the content of Palmatine hydrochloride (C_21_H_21_NO_4_-HCl) in the active ingredients of *Fibraurea recisa* Pierre was not less than 2.0%. The active component of *Pueraria montana* var. *lobata* (Willd.) Maesen and S.M.Almeida ex Sanjappa & Predeep (C_21_H_20_O_9_) is at least 2.4%. In addition, the extraction process of medicinal materials is fixed, which ensures the stability and repeatability of pharmacological action of compounds to a certain extent (Table 2). Secondly, we have studied the main chemical composition of the individual botanicals in the YJSB (Table 3).

**TABLE 2:** Quality standards of the YJSB.

Drug name	Active ingredients	Control standard
Arundinae herba	water extract	10% minimum
Dregeae radix	95% ethanol extract	10% minimum
Fibraureae caulis	Palmatine chloride	2% minimum
Puerariae lobatae radix	puerarin	2.4% minimum
Mappianthuse caulis	water extract	7% minimum
Anodendrone caulis	95% ethanol extract	16% minimum
Clerodendrume radix	95% ethanol extract	5% minimum
Glycyrrhizae radix et rhizoma	Liquiritin, glycyrrhizic acid	0.5%, 2% minimum

**TABLE 3:** Profile of main chemical composition.

Species name	Compound	References
Arundina graminifolia (D.Don) Hochr	lusianthridin	Liu et al. (2017), Xingyu et al. (2021)
	rhapontigen	Liu et al. (2017), Xingyu et al. (2021)
	p-hydroxytoluene ethyl ether	Liu et al. (2017), Xingyu et al. (2021)
	cucapitoside	Liu et al. (2017), Xingyu et al. (2021)
	dengibsin	Liu et al. (2017), Xingyu et al. (2021)
	1-(4-hydroxy-3,5-dimethoxyphenyl) propan-1-one	Liu et al. (2017), Xingyu et al. (2021)
*Dregea sinensis* Hemsl	n-carboxyl-2-hydroxy-4-pyrrole	Liao et al. (2016)
	Butanedioic aci,2-hydroxy-1,4-dibutylester	Liao et al. (2016)
	Propanoic acid,3-propoxy-butyl ester	Liao et al. (2016)
	propanoic acid,3-ethoxy-butyl ester	Liao et al. (2016)
	methyl shikimate	Liao et al. (2016)
	n-carboxyl-2-hydroxy-4-pyrrole	Liao et al. (2016)
*Fibraurea recisa* Pierre	stephanine	Bartley et al. (1994)
	roemarine	Rao et al. (2009)
	jatrorrhizine	Hussain et al. (1989)
	palmatine	Hussain et al. (1989)
	groenlandicine	Rao et al. (2009)
	berberine	Lenka et al. (2007)
*Pueraria montana* var. *lobata* (Willd.) Maesen and S.M.Almeida ex Sanjappa & Predeep	3′-hydroxypuerarin	Tungmunnithum et al. (2020), Hua and Chang (2022)
	puerarin	Tungmunnithum et al. (2020), Hua and Chang (2022)
	paidzin	Tungmunnithum et al. (2020), Hua and Chang (2022)
	formononetin	Tungmunnithum et al. (2020), Hua and Chang (2022)
	coumestrol	Tungmunnithum et al. (2020), Hua and Chang (2022)
	soyasapogenol A	Tungmunnithum et al. (2020), Hua and Chang (2022)
*Mappianthus iodoides* Hand. -Mazz	9-hydroxy-4,6-megastigmadien-3-one	Jiang et al. (2018)
	blumenol A	Jiang et al. (2018)
	9,10-dihydroxy-4,7-megastigmadi	Jiang et al. (2018)
	3,3-didemethoxyverrucosin	Jiang et al. (2018)
	mappine A	Xiao et al. (2011)
	mapposidic acid	Xiao et al. (2011)
*Anodendron nervosum*Kerr	3,4-dihydroxybenzaldehyde	Li et al. (2014)
	3,4-dihydroxybenzoic acid	Li et al. (2014)
	oleanolic acid	Li et al. (2014)
	iboluteine	Liu et al. (2019)
	venoter	Liu et al. (2019)
	lirofolines A	Liu et al. (2019)
*Clerodendrum chinense* (Osbeck) Mabb	octacosanoate taraxerol	Yue (2013)
	taraxerol	Yue (2013)
	myricadiol	Yue (2013)
	friedelin	Yue (2013)
	tetracosanoic acid	Yue (2013)
	indolyl-3-carboxylic acid	Yue (2013)
	elerodenoside A	Yue (2013)
*Glycyrrhiza uralensis* Fisch. ex DC	glycyrrhizic acid	Tan et al. (2022)
	diisobutyl phthalate	Tan et al. (2022)
	rutinum	Tan et al. (2022)
	liquiritin	Tan et al. (2022)
	phthalate	Tan et al. (2022)
	liquiritigenin	Tan et al. (2022)
	isoglycyrrhizol	Tan et al. (2022)
	scopoletin	Tan et al. (2022)

### 4.2 Determination of the content of jatrorrhizine and berberine in the blood of YJSB

**FIGURE 2:**
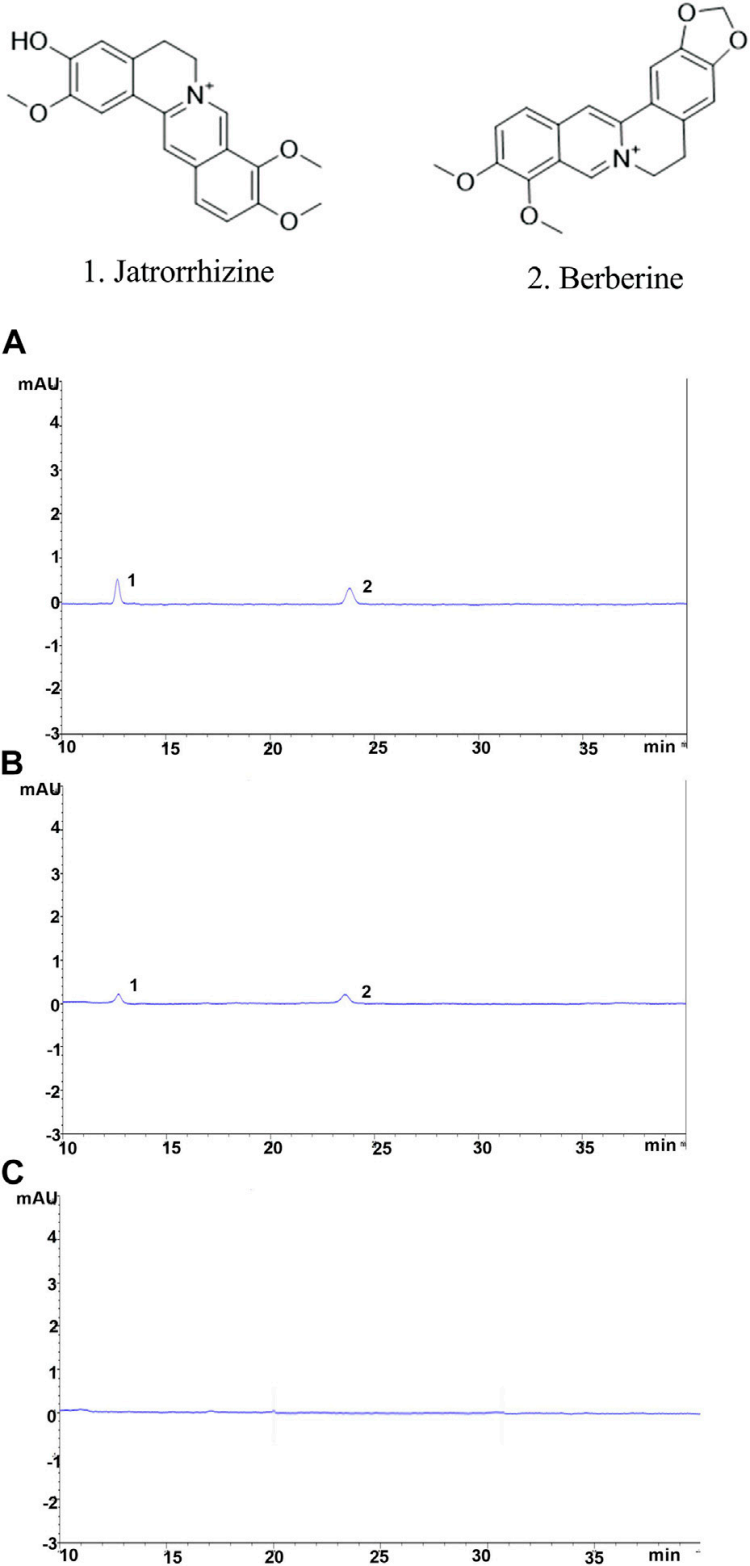
Chromatographic conditions and system suitability examination. **(A)** Jatrorrhizine and berberine mixed reference substance, **(B)** spectrum of the test substance, **(C)** spectrum of the normal serum; 1. Jatrorrhizine 2. Berberine.

The following are the results of the YJSB jatrorrhizine and berberine content determinations: 3 batches of SD rats were collected, treated according to method 2.1, and fed into the sample for analysis. Jatrorrhizine and berberine had retention times of 12.6 and 23.5 min, respectively. The separation degree R was greater than 1.5, and the number of trays was not less than 10000. The control and test products of jatrorrhizine and berberine exhibited good separation effects, and there was no interference in the corresponding position compared to normal serum. These results indicated that the chromatographic conditions could be employed to determine jatrorrhizine and berberine (Figure 2). The relative standard deviation (RSD) values of jatrorrhizine and berberine were 0.249% and 0.383%, respectively (n = 6). The levels of jatrorrhizine and berberine in the three batches of samples were determined (Table 4).

**TABLE 4:** Sample content measurement results.

Groups	Jatrorrhizine content (ng/mL)	Berberine content (ng/mL)
1	15.3	18.6
2	14.8	18.4
3	15.6	18.5

### 4.3 YJSB improved the general condition of hepatic fibrosis in rats

During the experiment, the rats in the normal exhibited normal hair color and quality and normal food intake and activity. On the other hand, the rats in the model had rough fur, bulging on both sides of the abdomen, and behaviors such as shrugging, loose disarray, and reduced food intake and activity, such as mental depression and huddling. All of the symptoms above improved in the rats receiving treatment. In the model, the surface of liver tissue was uneven and hard, with noticeable white nodule-like alterations, indicating severe liver injury. Following treatment, the CCl_4_-induced liver fibrosis was significantly inhibited, the surface of the liver was flattened and softer, and the area covered by white nodules was reduced.

### 4.4 YJSB improved the inflammatory infiltration of liver tissue in rats with hepatic fibrosis

**FIGURE 3:**
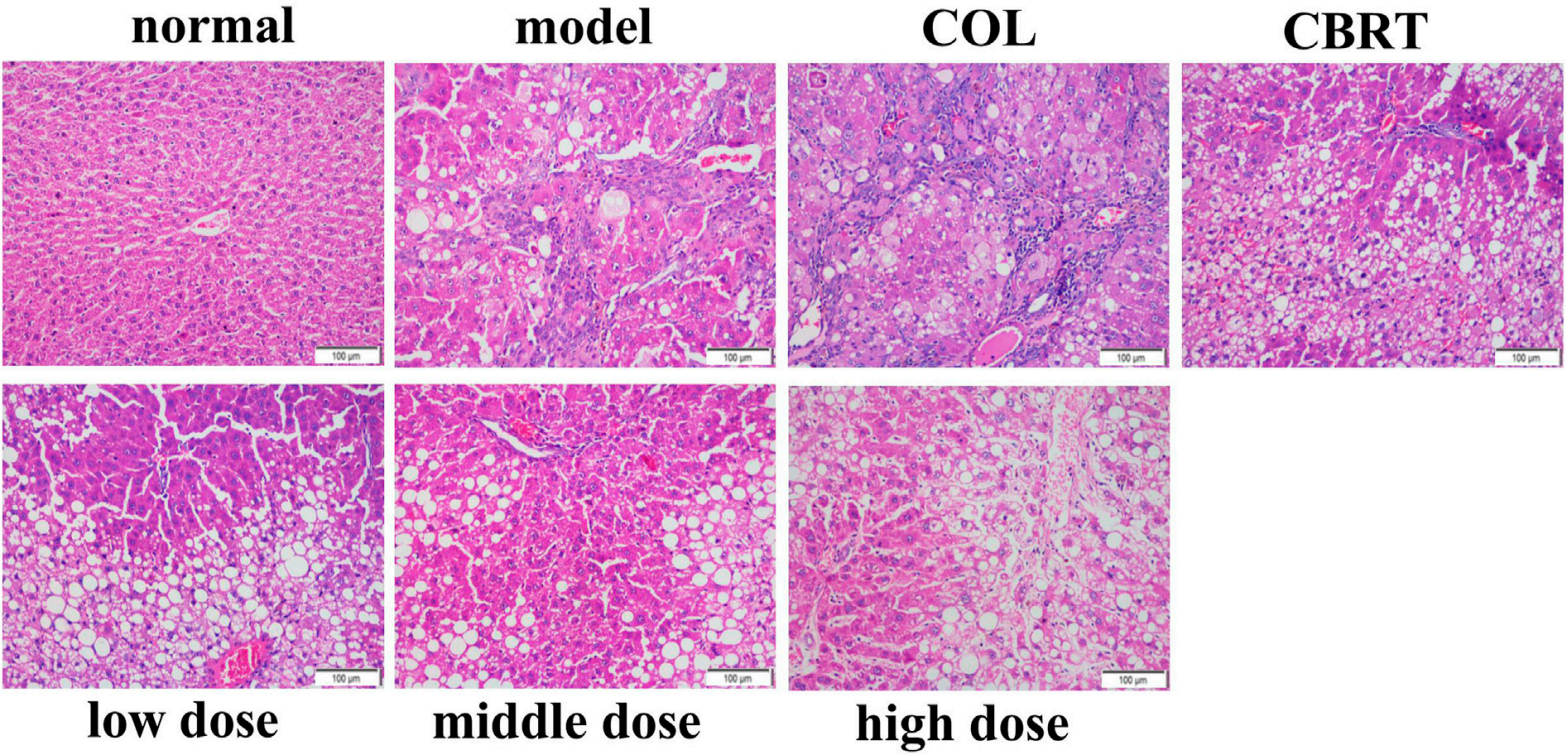
YJSB improved the inflammatory infiltration of liver tissue in rats with hepatic fibrosis. H&E staining of the rat liver (200×) (n = 3).

H&E staining could be employed to observe liver inflammation and changes in liver cell structure. In the normal, the structure of hepatic lobules was complete, and the cords of hepatic cells were mostly arranged in a radial pattern around the central vein (Figure 3). There was no expansion, occlusion, or distortion of hepatic sinuses and no obvious proliferation of hepatocytes, inflammation, and necrosis (Figure 3). In comparison to the normal, the model showed varying degrees of inflammatory cell infiltration in the portal area (Figure 3). Some of the normal liver structures were damaged, with severe disintegration and necrosis, moderate or severe fibrous tissue hyperplasia, and some obvious false lobules (Figure 3). The liver of rats in the treatment exhibited mild inflammatory cell invasion, edema, and necrosis of the liver cells, as well as the reduced proliferation of hepatic lobules and fibrous tissue and improved liver structure compared to the model (Figure 3). These results suggested that YJSB could protect the liver by reducing inflammatory invasion and enhancing liver structure.

### 4.5 YJSB reduced the deposition of collagen fibers in the liver tissue of rats with hepatic fibrosis

**FIGURE 4:**
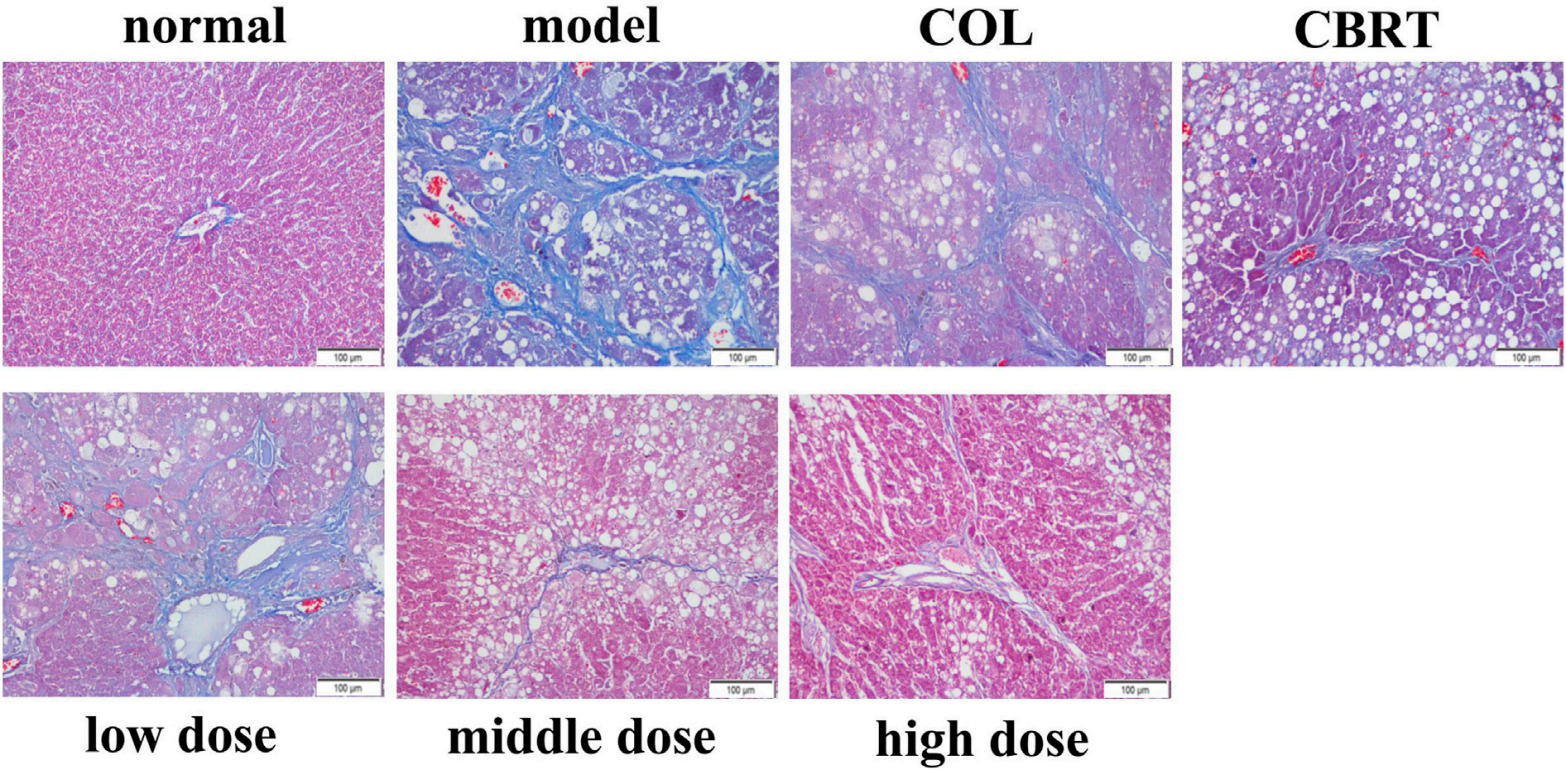
YJSB reduced the deposition of collagen fibers in the liver tissue of rats with hepatic fibrosis. Masson’s trachoma staining of the rat liver (200×) (n = 3).

The expression of collagen fibers could be observed using Masson’s trachoma staining. In the normal, the structure of hepatic lobules was clear, and only a minor amount of collagen fiber hyperplasia was observed in the portal region (tube wall) (Figure 4). Besides, there was no evident inflammatory cell invasion, edema, and hyperemia (Figure 4). The structure of hepatic lobules in the model was disorganized, and many inflammatory cells invaded the central vein and the portal area compared to the normal (Figure 4). There was significant collagen fiber proliferation with increased collagen fiber expression on the surface of liver tissue (Figure 4); the portal region was visible with some false lobules formed and some significant large areas exhibited in blue (Figure 4). The liver inflammatory cell invasion, collagen fiber expression, and false lobule formation were decreased in the treatment compared to the model (Figure 4). These results suggested that YJSB could protect the liver by reducing the deposition of collagen fibers.

### 4.6 YJSB reduced the deposition of reticular fibers in the liver tissue of rats with hepatic fibrosis

**FIGURE 5:**
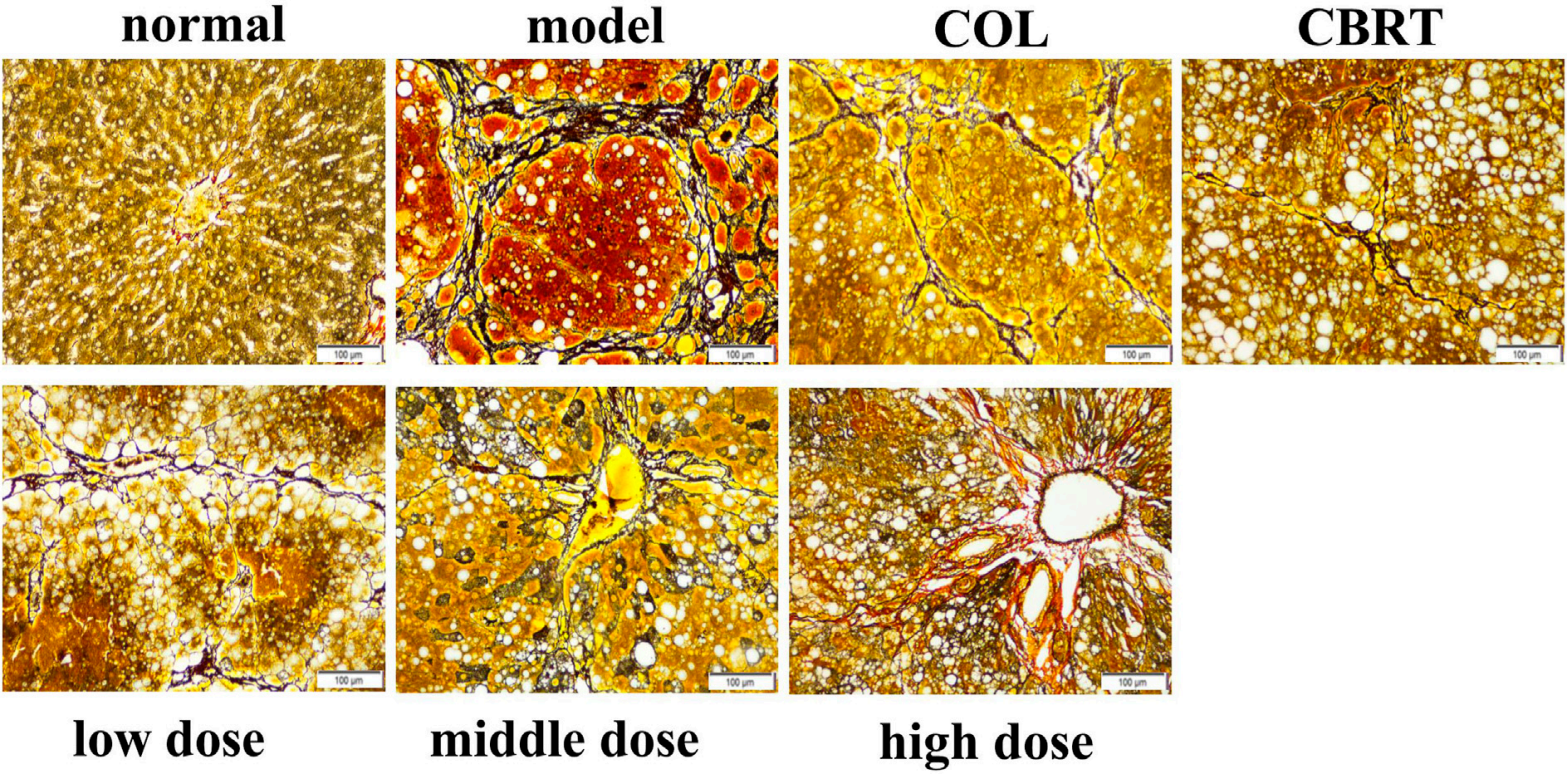
YJSB reduced the deposition of reticular fibers in the liver tissue of rats with hepatic fibrosis. Ag staining of the rat liver (200×) (n = 3).

Ag staining could be employed to observe the deposition of reticular fibers. The structure of the hepatic lobules was clear, and the reticular scaffolds were intact in the normal. There was no evidence of inflammatory cell invasion or reticular fibrosis (Figure 5). Compared to the normal, the model’s hepatic lobule structure was disorganized. The reticular scaffolds were damaged, particularly in the collagenous hyperplasia region of the portal and bridge-like necrosis areas (Figure 5). The hepatic reticular fibrous tissue proliferation was reduced in the treatment in comparison to the model (Figure 5); the fibrous tissue was dispersed around the liver cells, and the formation of false lobules was reduced (Figure 5). According to the results, YJSB protects the liver by reducing the deposition of reticular fibers.

### 4.7 YJSB improved the liver function in rats with hepatic fibrosis

**FIGURE 6:**
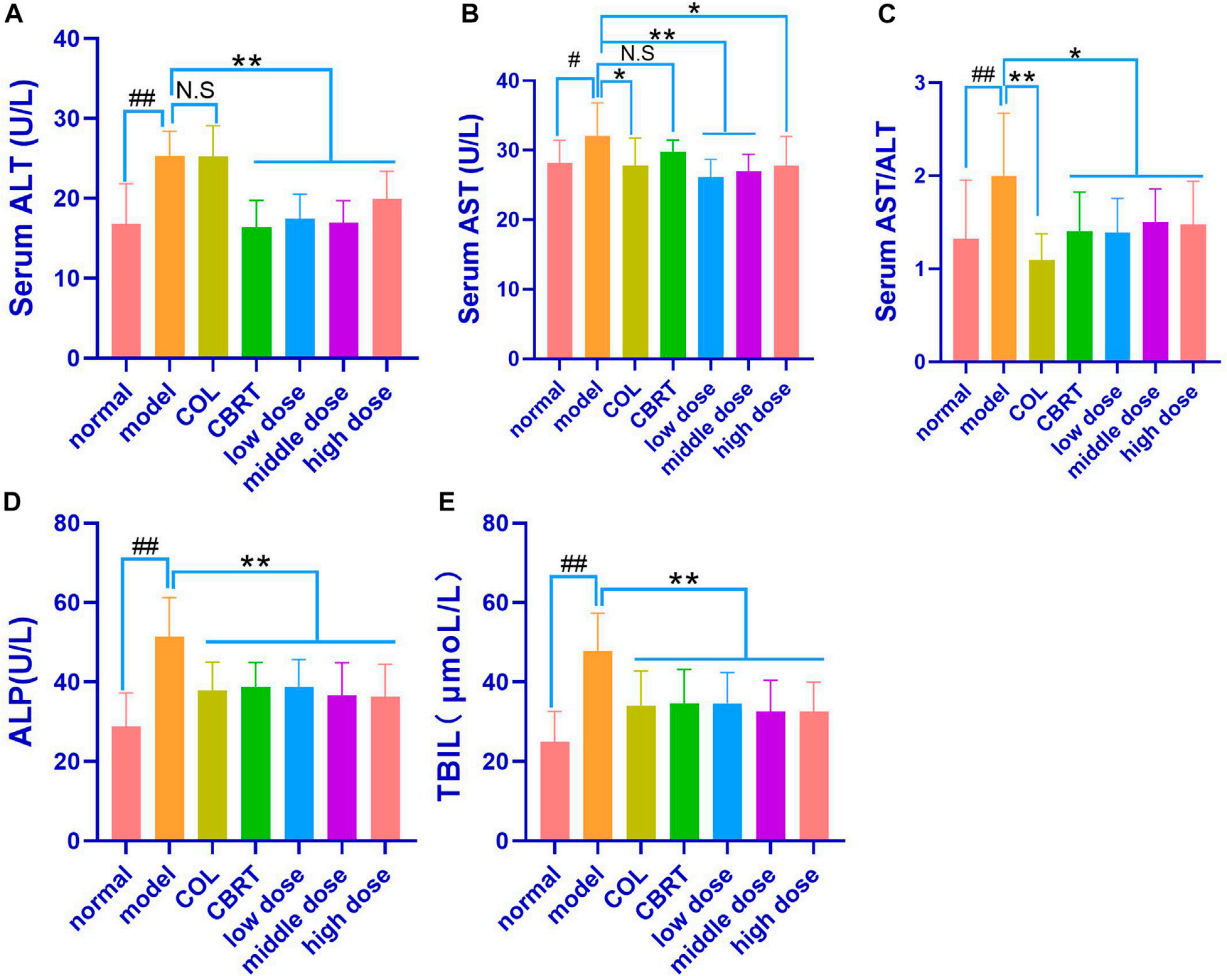
YJSB improved liver function in rats with hepatic fibrosis. **(A–E)** The levels of serum ALT, AST, AST/ALT, ALP, and TBIL after the 4-week drug intervention. (n = 8). *#p* < 0.05, *##p* < 0.01 vs. normal; ^*^ *p* < 0.05, ^**^ *p* < 0.01, No statistical significance (NS): *p* > 0.05 vs. model.

We measured serum ALT, AST, AST/ALT, ALP, and TBIL levels to investigate CCl_4_-induced hepatocyte damage. As shown in Figure 5, a significantly enhanced level of ALT, AST, AST/ALT, ALP, and TBIL in the blood was observed in rats treated with CCl_4_ (50.87%, 13.66%, 51.03%, 78.75%, and 92.00%, respectively) as compared to normal (*p* < 0.01, *p* < 0.05; Figures 6A–E). When compared to the model, COL therapy resulted in significantly lower (*p* < 0.01, *p* < 0.05; Figures 6A–E) AST (13.26%), AST/ALT (45.06%), ALP (26.39%), and TBIL (28.90%) values in the blood. When compared to the model, CBRT therapy resulted in significantly lower (*p* < 0.01, *p* < 0.05; Figures 6A–E) ALT (35.37%), AST/ALT (29.61%), ALP (24.63%), and TBIL (27.57%) values in the blood. In rats, treatment with YJSB at low, medium, and high doses resulted in significantly lower (*p* < 0.01, *p* < 0.05; Figures 6A–E) ALT (31.01%, 33.21%, and 21.13%, respectively), AST (18.45%, 15.83%, and 13.31%, respectively), AST/ALT (30.41%, 24.61%, and 26.21%, respectively), ALP (24.76%, 28.92%, and 29.50%, respectively) and TBIL (27.58%, 32.09% and 32.01%, respectively) values in the blood as compared to the model. The results showed that YJSB had a protective effect on liver function.

### 4.8 YJSB reduced the contents of HA, PIIINP, ColIV, and LN in the serum of rats with hepatic fibrosis

**FIGURE 7:**
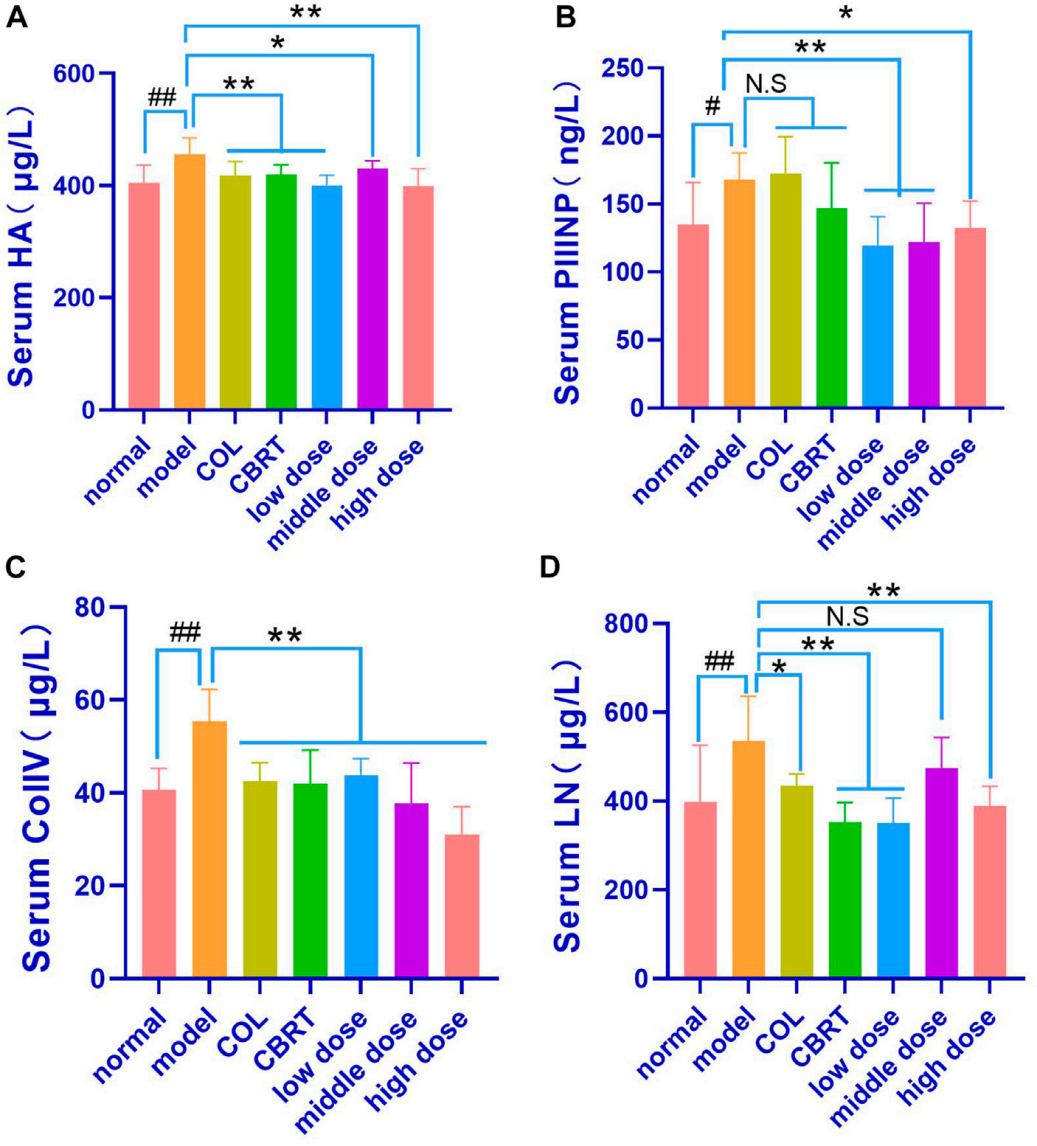
YJSB reduced the levels of HA, PIIINP, ColIV, and LN in the serum of rats with hepatic fibrosis. **(A–D)** The levels of serum HA, PIIINP, ColIV, and LN after the 4-week drug intervention. (HA, PIIINP, and ColIV: n = 8; LN: n = 6). ^*#*^ *p* < 0.05, ^*##*^ *p* < 0.01 vs. normal; ^*^ *p* < 0.05, ^**^ *p* < 0.01, N.S: *p* > 0.05 vs. model.

When compared to the control, continuous exposure to CCl_4_ caused a significant increase (*p* < 0.01, *p* < 0.05; Figures 7A–D) in HA (1.13-fold), PIIINP (1.25-fold), ColIV (1.36-fold), and LN (1.35-fold). The contents of HA (8.43% and 7.96%, respectively), ColIV (23.31% and 24.31%, respectively), and LN (18.96% and 34.32%, respectively) in the Col and CBRT were significantly decreased (*p* < 0.01, *p* < 0.05; Figures 7A, C, D). The contents of HA (12.17%, 5.79%, and 12.65%, respectively), PIIINP (28.77%, 27.39%, and 21.16%, respectively), and ColIV (20.95%, 31.95%, and 44.16%, respectively) were significantly decreased (*p* < 0.01, *p* < 0.05; Figures 7A–C) in the YJSB low-dose, middle-dose and high-dose, and the content of LN (34.55% and 27.26%, respectively) were significantly lower (*p* < 0.01; Figure 7D) in the YJSB low-dose and high-dose than that in the model. According to the findings, YJSB could significantly improve serum hepatic fibrosis indicators such as HA, PIIINP, ColIV, and LN.

### 4.9 YJSB reduced the contents of Hyp and TGF-β1 in the serum of model rats

**FIGURE 8:**
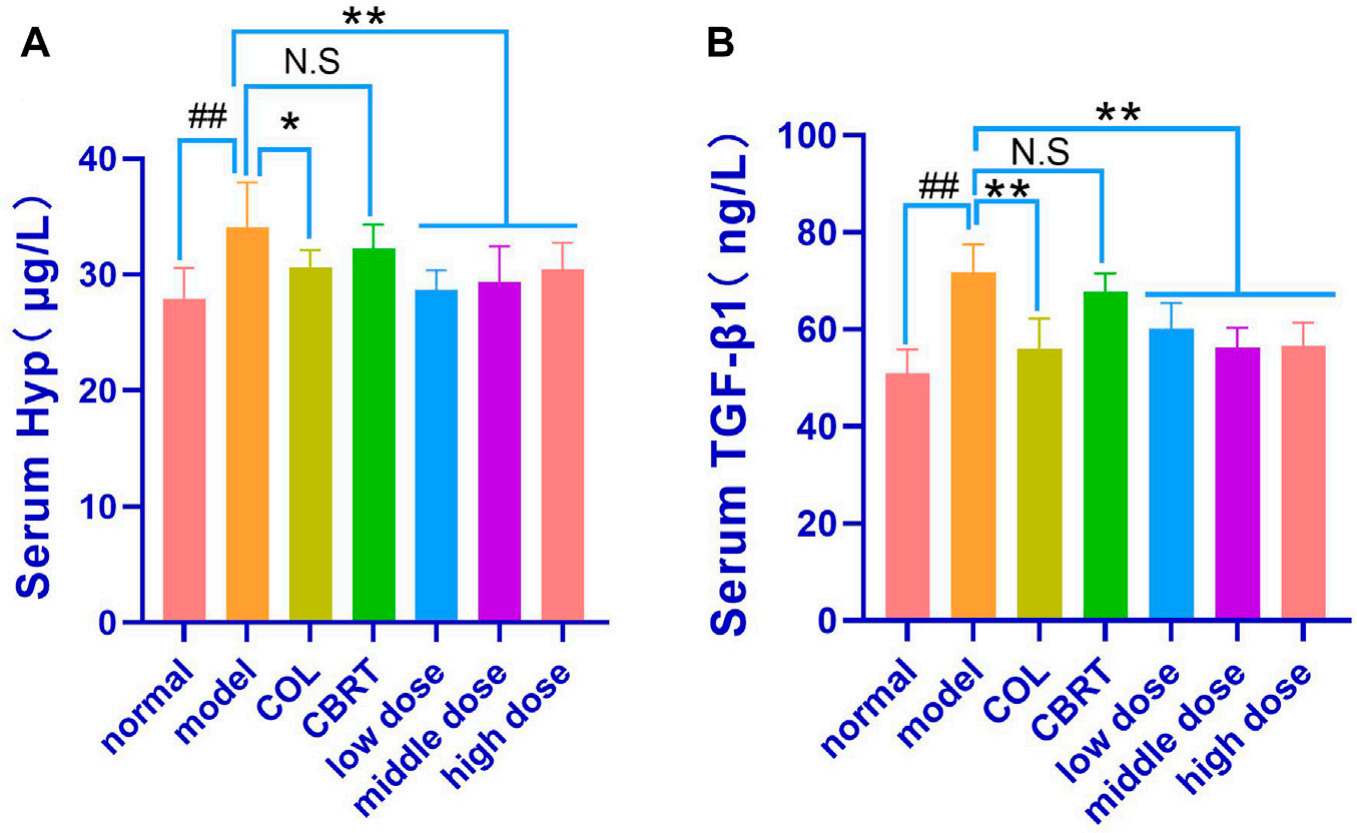
YJSB reduced the contents of Hyp and TGF-β1 in the serum of model rats. **(A,B)** The levels of serum Hyp and TGF-β1 after the 4-week drug intervention. (n = 8). ^#^ *p* < 0.05, ^##^ *p* < 0.01 vs. Normal; *p < 0.05, **p < 0.01, NS: *p* > 0.05 vs. model.

We measured serum Hyp and TGF-β1 levels because TGF-β1 is an essential pro-fibrotic mediator, and Hyp is a recognized marker of collagen accumulation in the liver (Lu et al., 2019). The results indicated that the contents of Hyp (21.98%) and TGF-β1 (40.82%) had significantly increased (*p* < 0.01, *p* < 0.05; Figures 8A, B) in the model when compared to normal. In contrast, therapy with COL, YJSB low-dose, middle-dose, and high-dose in rats caused a significant decrease (*p* < 0.01; Figures 8A, B) in Hyp (10.14%, 15.70%, 13.85%, and 10.51%, respectively) and TGF-β1 (21.96%, 16.19%, 21.66% and 21.23%, respectively) values in the liver homogenate as compared to the model. According to the results, YJSB could significantly improve the serum indicators of hepatic fibrosis, such as Hyp and TGF-β1.

### 4.10 YJSB affected the release of oxidative stress factors (SOD and MDA)

**FIGURE 9:**
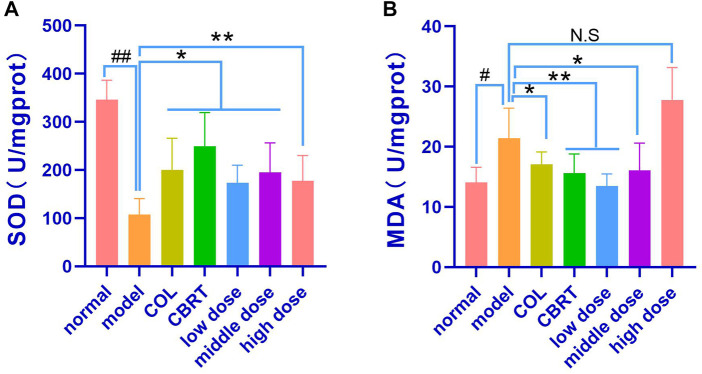
YJSB affected the release of oxidative stress factors. **(A,B)** The levels of liver homogenate SOD and MDA after the 4-week drug intervention. (SOD: n = 6; MDA: n = 6). #*p* < 0.05, ##*p* < 0.01 vs. normal; *p < 0.05, **p < 0.01, NS: *p* > 0.05 vs. model.

MDA may indirectly reflect the degree of oxidative stress injury to cells, whereas SOD may protect cells from oxidative stress and rapidly improve and restore the condition of damaged cells (Sha et al., 2016; Margherita et al., 2021), SOD and MDA levels in liver homogenate were measured. The experimental results revealed that, when compared to normal, the model’s liver homogenate contained significantly less SOD (14.79%) and significantly more MDA (34.45%) (*p* < 0.01, *p* < 0.05; Figures 9A, B). In the liver homogenates of the Col, CBRT, YJSB low-dose, middle-dose, and high-dose, the content of SOD (18.40%, 16.09%, 20.49%, 17.27%, and 22.49%, respectively) increased significantly (*p* < 0.01, *p* < 0.05; Figure 9A), while the content of MDA (16.44%, 47.31%, 31.00%, and 21.71%, respectively) in the liver homogenate of the Col, CBRT, YJSB low-dose and middle-dose decreased significantly (*p* < 0.01, *p* < 0.05; Figure 9B). According to the findings, YJSB may stimulate the release of oxidative stress factors.

### 4.11 YJSB promoted the expression of GCLC, GCLM, NQO1, and HO-1 in the Keap1-Nrf2 pathway

**FIGURE 10:**
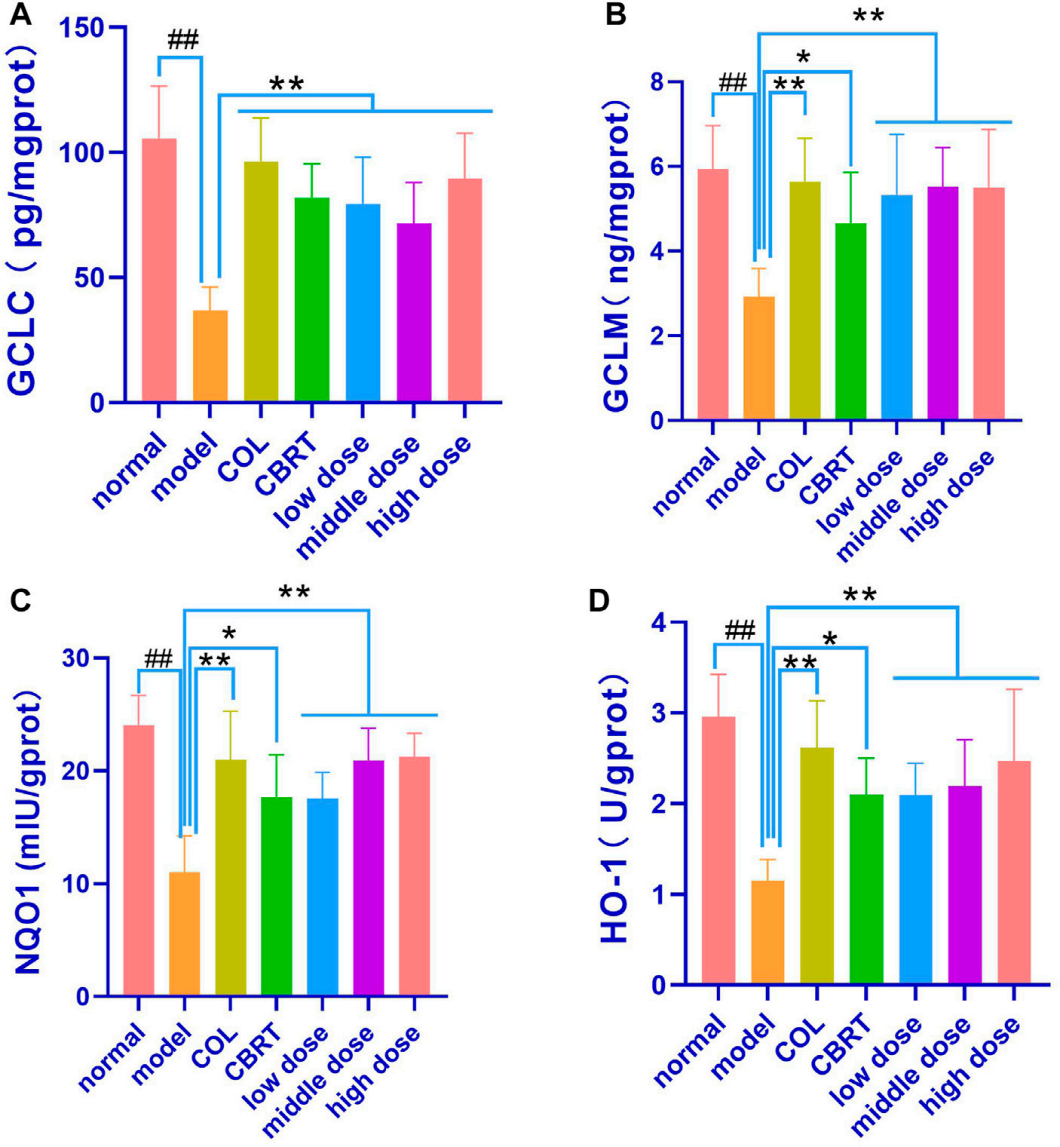
YJSB promoted the expression of GCLC, GCLM, NQO1, and HO-1 in Keap1-Nrf2 pathway. **(A–D)** The levels of liver homogenate GCLC, GCLM, NQO1, and HO-1 after the 4-week drug intervention. (n = 8). ^#^ *p* < 0.05, ^##^ *p* < 0.01 vs. Normal; ^*^ *p* < 0.05, ^**^ *p* < 0.01, NS: *p* > 0.05 vs. model.

To investigate the relationship between GCLC, GCLM, NQO 1, and HO-1 and Keap 1-Nrf 2, we measured GCLC, GCLM, NQO 1, and HO-1 in liver homogenate. The results demonstrated that the contents of GCLC (56.13%), GCLM (45.53%), NQO1 (38.63%), and HO-1 (34.69%) in the liver homogenates were significantly low (*p* < 0.01, *p* < 0.05; Figures 10A–D) in the model when compared to normal. GCLC (1.92-fold, 1.39-fold, 1.52-fold, 1.50-fold, and 1.80-fold, respectively), GCLM (1.52-fold, 1.19-fold, 1.40-fold, 1.54-fold and 1.40-fold, respectively), NQO1 (1.51-fold, 1.07-fold, 1.31-fold, 1.32-fold and 1.44-fold, respectively), HO-1 (1.44-fold, 1.13-fold, 1.25-fold, 1.32-fold and 1.38-fold, respectively) content in the liver homogenate of the COL, CBRT, YJSB low-does, middle-does, and high-dose were significantly increased (*p* < 0.01; Figures 10A–D) in compared to the model. According to the results, YJSB could promote the expression of GCLC, GCLM, NQO1, and HO-1 in the Keap1-Nrf2 pathway.

### 4.12 YJSB regulated the expression of Keap1 and Nrf2 proteins in the Keap1-Nrf2 pathway

**FIGURE 11:**
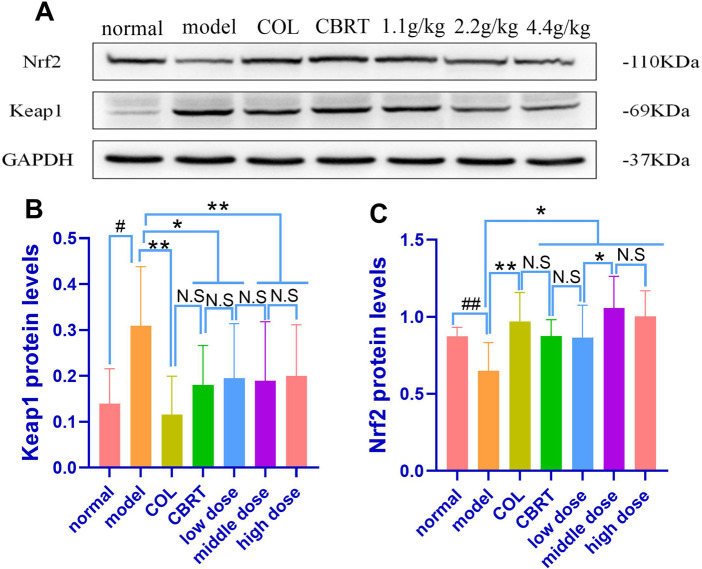
The protein expression of Nrf2 and Keap1. **(A–C)** Relative protein levels of Nrf2 and Keap1 in the liver of hepatic fibrosis rats. (n = 8). #*p* < 0.05, ##*p* < 0.01 vs. normal; *p < 0.05, **p < 0.01 vs. model.

We performed experimental validation of the Keap1 and Nrf2 proteins to investigate the underlying mechanisms of YJSB against mechanisms that cause CCl_4_-induced liver fibrosis. When compared to normal, the experimental results showed that Nrf2 (25.75%; *p* < 0.01; Figures 11A, B) protein expression had significantly slowed, and Keap1 (2.21-fold; *p* < 0.01; Figures 11A, C) protein expression had significantly increased. When compared to the model, Nrf2 (1.49-fold, 1.35-fold, 1.33-fold, 1.63-fold, and 1.55-fold, respectively) protein expression in the COL, CBRT, YJSB low-does, middle-does, and high-dose was significantly increased (*p* < 0.01, *p* < 0.05; Figures 11A, B), while Keap1 (62.54%, 41.69%, 37.12%, 38.92%, and 35.53%, respectively) protein expression was significantly decreased (*p* < 0.01, *p* < 0.05; Figures 11A, C). The findings suggest that YJSB can regulate the expression of Keap1 and Nrf2 proteins in the Keap1-Nrf2 pathway.

### 4.13 YJSB promotes Nrf2 protein to enter the nucleus

Given the importance of Nrf 2 in the liver, we used fluorescence immunohistochemistry to examine Nrf 2 expression in liver tissue.

**FIGURE 12:**
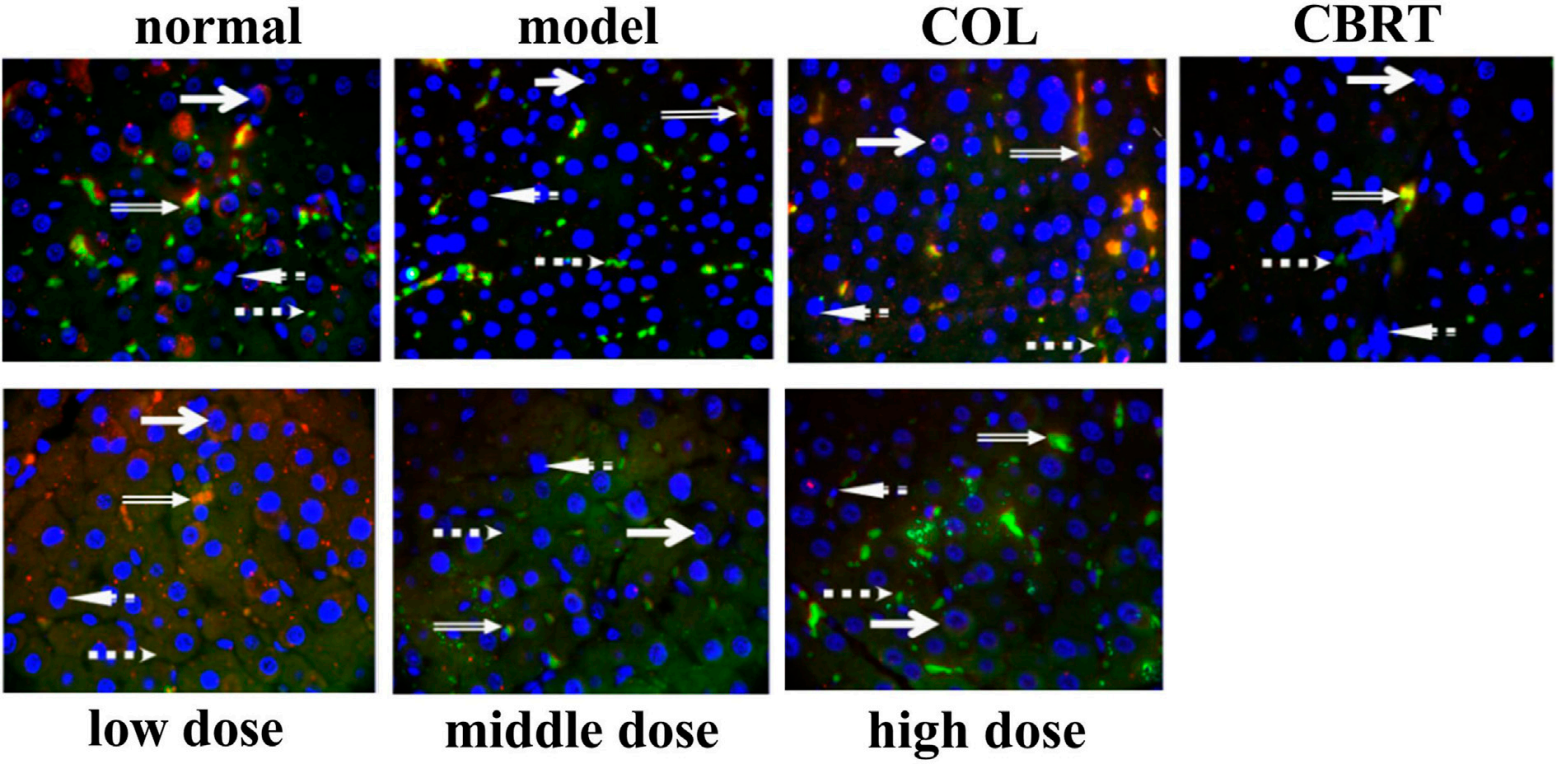
Results of fluorescence immunoassay of Keap1 and Nrf2 proteins in liver tissue (1,000×). Red: Nrf2 protein; Green: Keap1 protein; Blue: 4′, 6-diamidino-2-phenylindole (DAPI); Orange: The binding state of Keap1-Nrf2 (n = 3).

Fluorescence immunohistochemistry revealed that the nuclei were blue and irregularly shaped. In the cytoplasm, the positive expression of Keap1 was green, and that of Nrf2 was red (Figure 12). When Nrf2 entered the nucleus, it overlapped red and blue, and there was a shadow in the irregular blue circle of the nucleus (Figure 12). The Nrf2 expression and Nrf2 protein entry into the nucleus decreased in the model, while the Keap1 expression signal was stronger than normal (Figure 12). When compared to model, Keap1 expression was decreased, and the signal was weaker in the treatment group (Figure 12). In the treatment group, both the expression of Nrf2 and the number of nuclei entered increased (Figure 12). According to the findings, YJSB may promote the entry of the Nrf2 protein into the nucleus.

## 5 Discussion

The continuous accumulation of experience of the Dai people in the fight against diseases might be summed up as Dai medicine. It has absorbed the experience of ancient Indian and traditional Chinese medicine and possesses distinctive national and local characteristics (Zhen et al., 2013). The Yunnan Dai people of China are rich in medical resources with a wide range of clinical applications, featuring good curative effects and a few adverse effects. Dai medical compound YJSB is an effective substance in treating various liver diseases (Yang et al., 2022). Hepatic fibrosis is a progressive disease with a long course (sometimes decades), which could lead to cirrhosis and liver cancer (Ge et al., 2021). The prevention and treatment of hepatic fibrosis is a common challenge faced by hundreds of millions of patients with the chronic liver disease worldwide. The role of YJSB has a therapeutic effect. We first gave CCl_4_ for 6 weeks to replicate the liver fibrosis animal model, and then we gave YJSB for 4 weeks to treat liver fibrosis rats.

YJSB is a hospital preparation developed by Xishuangbanna Dai Hospital in Yunnan, China, made into capsules (also known as Baijie Capsules) and used in hospitals for many years (Xiaohua et al., 2015; Yang et al., 2022). In a previous study, mice were given 88 g/kg in a single gavage and were found to be in good general condition 14 days later with no mortality. The findings above indicate that the toxicity of YJSB is low (Wang, 2013). The doses of YJSB in our present study were 1.1 g/kg, 2.2 g/kg, and 4.4 g/kg, of which 2.2 g/kg was close to the dose of YJSB commonly used clinically converted to rats. No behavioral changes of toxicity were found in the animals during the course of the experiment. After the experiment, the animals were executed, the organs were examined, and no abnormal changes were found in the organs, indicating that the doses in this study were safe.

Although the liver has a significant capacity for regeneration, excessive regeneration might result in fibrosis (Hernandez-Gea and Friedman, 2011). All chronic liver diseases exhibit abnormal regeneration as a common pathological change, and possible sources of myofibroblasts following a chronic liver injury include portal vein fibroblasts, mesenchymal cells, and fibroblasts (Hernandez-Gea and Friedman, 2011). Hepatic stellate cells are unique mesenchymal cells of the liver (Blaner et al., 2008) that normally exist in a static state and accumulate vitamin A. When oxidative stress damages occur in the body, hepatic stellate cells could be activated to mediate various signaling pathways and lead to cell morphologies similar to myoblast fiber changes. This could result in excessive deposition of collagen-dominated ECM and promote the occurrence and development of hepatic fibrosis (Özlem, 2014; Young et al., 2020).

Increasing evidence reveals that the Keap1-Nrf2 pathway is the most important endogenous antioxidant pathway thus far (Yang et al., 2014). When at rest, Nrf2 conjugates with its molecular partner Keap1 to form a stable dimer that resides in the cytoplasm in an inactive state. When a cell is stimulated by oxidative stress or other stimuli, Nrf2 is activated, dissociates from the dimer after separating from Keap1, and undergoes nuclear transfer; it then enters the nucleus and binds with the antioxidant reaction element (ARE) to induce and regulate target genes. Protein expressions of GCLC, GCLM, NQO1, and HO-1 were increased, thus, enhancing the antioxidant capacity of cells, increasing the concentration of antioxidant stress factor SOD, and reducing the release of oxidative stress factor MDA (Faju et al., 2021). According to an earlier study (Truong et al., 2014), activation of the Keap1-Nrf2 pathway could inhibit the activation of hepatic stellate cells and their production of a large amount of collagen, thus, inhibiting the occurrence and development of liver fibrosis.

In our study, we first established a model of a rat with hepatic fibrosis; CCl_4_-induced animal hepatic fibrosis was similar to human hepatic fibrosis in most aspects of morphology and pathophysiology (Shaban et al., 2021). Pathological slides are the gold standard of disease. Our results confirmed the successful replication of the hepatic fibrosis model and the drug’s effect on liver tissue at the cellular level. H&E, Ag, and Masson’s trichrome staining revealed a trend of liver inflammation, structural changes in liver cells, increased collagen fiber accumulation, and excessive reticular fiber deposition in the model group. YJSB treatment, on the other hand, significantly reduced hepatic inflammatory response, improved hepatic cell structural changes, and reduced collagen fiber and reticular fiber deposition, possibly due to its ability to inhibit hepatic stellate cell activation.

We also demonstrated the efficacy of YJSB in the protection of the liver. ALT and AST as liver-specific biomarkers could be employed to evaluate the degree of liver injury, and their activity is positively correlated with liver injury (Behnaz et al., 2021). CCl_4_-induced rat hepatic fibrosis model could cause acute liver cell injury, increase cell membrane permeability, and release a significant quantity of ALT, AST, ALP, and TBIL into the blood (Tohru et al., 2005). Clinically, the increase in AST activity is believed to be greater than ALT, indicating the onset of chronic and advanced stages of liver disease (Karim et al., 2015). The ALT, AST, ALP, and TBIL results indicated that the CCl_4_-induced hepatic fibrosis model had progressed to a chronic and progressive state at the end of this experiment, consistent with the characteristics of hepatic fibrosis progression. Meanwhile, studies (Behnaz et al., 2021) have demonstrated that HA is one of the matrix components synthesized by mesenchymal cells. HA could accurately and sensitively represent the amount of generated fibers in the liver and the liver cells’ damage status compared to other liver detection indexes. LN is a unique non-collagenous structural protein in the basement membrane, predominantly derived from hepatic stellate cells, which could reflect the degree of hepatic fibrosis activity and portal vein pressure. The synthesis status of hepatic fiber and inflammatory activity could be reflected by PCIIINP. ColIV, as the principal component of the basement membrane, represents the collagen renewal rate of the basement membrane and could sensitively reflect the process of liver fibrosis, which is one of the earliest symptoms of liver fibrosis. The levels of HA, LN, PCIIINP, and ColIV in the model group were found to be proportional to the levels of ALT, AST, ALP, and TBIL. YJSB reduced HA, LN, PCIIINP, ColIV, ALT, and AST levels in rat serum, indicating that YJSB had a protective effect on injured liver cells and could improve liver fibrosis. It is likely that CCl_4_ causes ECM molecules to form and accumulate, increasing fiber volume, whereas medications could effectively reduce HA, LN, PCIIINP, and ColIV concentrations in serum, preventing the development of hepatic fibrosis.

Second, the activation of hepatic stellate cells by cytokines and other mediators is considered to be an important event in the pathophysiology of liver fibrosis and activated hepatic stellate cells or myofibroblasts are the primary sources of ECM molecules, including collagen, non-collagen glycoprotein, proteoglycan, and glycosaminoglycan (Chhimwal et al., 2020). TGF-β1 has been shown to be an important pro-fibrotic mediator. Hyp has been shown to be a recognized marker of collagen accumulation in the liver (Lu et al., 2019), playing an important role in the activation of hepatic stellate cells, as well as restricting hepatocyte proliferative response and increasing extracellular matrix protein production during hepatic repair (Piotrowska-Kempisty et al., 2020). Our results suggest that the levels of TGF-β1 and Hyp in the model group were significantly increased, promoting the occurrence of liver fibrosis. At the same time, YJSB could effectively reduce the levels of both and prevent hepatic fibrosis.

In addition, reduced fibrosis might be involved in oxidative stress factors and the Keap1-Nrf2 pathway (Alkhalifah et al., 2022). The content of MDA, an end product of lipid oxidation, could indirectly reflect the degree of oxidative stress injury of cells. SOD, an important antioxidant enzyme, could protect cells from oxidative stress and rapidly improve and restore the condition of damaged cells (Sha et al., 2016; Margherita et al., 2021). The Keap1-Nrf2 signaling pathway plays an important role and regulates GCLC, GCLM, NQO1, and HO-1 at various levels, which is approximately related to oxidative stress (Wenge and Ah-Ng, 2009). Our findings show that YJSB can lower MDA levels while increasing SOD levels in liver homogenates, implying that YJSB can act as an antioxidant stress factor, reducing the degree of liver damage caused by oxidative stress and protecting the liver. Simultaneously, the expressions of GCLC, GCLM, NQO1, and HO-1 in the liver of the model group were significantly decreased. In contrast, the expressions of GCLC, GCLM, NQO1, and HO-1 in the liver of the model group were significantly increased by YJSB, suggesting that YJSB might improve the antioxidant capacity of the redox system in rats, reduce oxidative stress, and interfere with the development of hepatic fibrosis.

Finally, in the CCL_4_-induced rat hepatic fibrosis model, the YJSB drug group activated the Keap1-Nrf2 signaling pathway, resulting in Keap1 release in the cytoplasm and Nrf2 entry into the nucleus to initiate a series of antioxidant reactions and promote the generation of antioxidant proteins. In the results of WB, the expression of Nrf2 in the administration group was higher in comparison to the normal group, which might be because the modification of Keap1 cysteine residues in the administration group was improved, which further promoted the dissociation of Keap1-Nrf2 and increased the amount of Nrf2 that entered the nucleus to combat oxidative stress.

In summary, our study suggests that YJSB could significantly protect rats against CCL_4_-induced hepatic fibrosis by alleviating liver injury and inhibiting the activation of hepatic stellate cells. The mechanism might be related to its ability to activate the Keap1-Nrf2 signaling pathway, regulate oxidative stress response, and play an anti-hepatic fibrosis role. Because YJSB protects the liver, it could have significant clinical implications. However, none of these studies have directly demonstrated that YJSB inhibits the activity of hepatic stellate cells, and its molecular basis is complex, necessitating further investigation.

## Data Availability

The original contributions presented in the study are included in the article/Supplementary Material, further inquiries can be directed to the corresponding author.

## Abbreviations

CBRT, Compound Biejia-Ruangan tablet; COL, Colchicine; Col Ⅳ, Collage Type-Ⅳ; CCL4, Carbon tetrachloride; ECM, extracellular matrix; GCLC, Glutamate cysteine ligase catalytic subunit; GCLM, Glutamate cysteine ligase modifier subunit; HA, Hyaluronic acid; HO-1, Heme Oxygenase 1; H&E, Hematoxylin and Eosin; Hyp, hydroxyproline; Keap1, Kelch-like 1; LN, Laminin; MDA, Malondialdehyde; NQO1, NAD(P)H: Quinone oxidoreductase; Nrf2, nuclear factor erythroid-2 related factor; PCIII, procollagen type-III; SOD, Superoxide dismutase; TGF-β1, Transforming growth factor-β1; WB, Western Blotting; YJSB, Yajieshaba.
