# Supplementary material for: Mechanisms of Yajieshaba in the treatment of liver fibrosis through the Keap1-Nrf2 signaling pathway
Source: Front Pharmacol. 2023 May 9;14:1124015. doi: 10.3389/fphar.2023.1124015 (PMC10203482; doi:10.3389/fphar.2023.1124015)
Supplement: Supplementary file 1 [file DataSheet1.PDF]

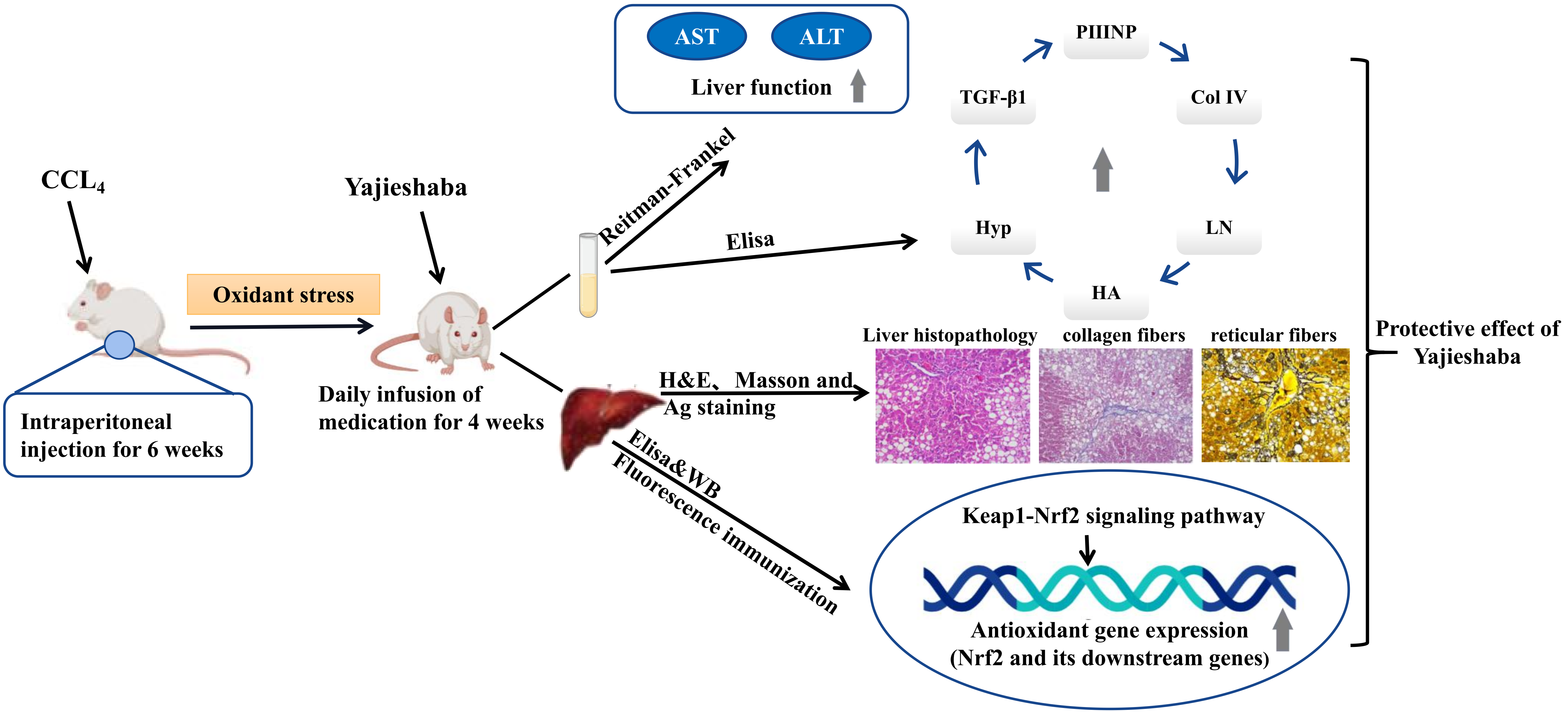

In order to test the hypothesis that Yajieshaba has a protective effect on hepatic fibrosis in rats through anti-oxidative stress. A rat model of hepatic fibrosis caused by CCL_4 was replicated, and the relevant indexes were detected by gavage of Yajieshaba . The results showed that Yajieshaba reduced oxidative stress injury and improved liver fibrosis by regulating the protein expression of GCLC, GCLM, NQO1 , HO-1 Keap1 and Nrf2 in the activated Keap1-Nrf2 pathway.
